# Anatomy and Topography of Coronary Sinus and Mitral Valve Annulus in Functional Mitral Regurgitation

**DOI:** 10.3389/fcvm.2022.868562

**Published:** 2022-04-22

**Authors:** Dennis Rottländer, Martin Saal, Alev Ögütcü, Hubertus Degen, Michael Haude

**Affiliations:** ^1^Department of Cardiology, Rheinlandklinikum Neuss, Neuss, Germany; ^2^Department of Cardiology, Faculty of Health, School of Medicine, University Witten/Herdecke, Witten, Germany; ^3^Department of Cardiology, Krankenhaus Porz am Rhein, Cologne, Germany

**Keywords:** mitral valve annulus, coronary sinus, CT-angiography, functional mitral valve regurgitation, indirect mitral valve annuloplasty

## Abstract

**Background:**

We aimed to investigate the anatomical relationship of the coronary sinus (CS) and the mitral valve annulus (MVA) in patients with or without functional mitral regurgitation (FMR) using a multislice CT (MSCT) software to determine (a) the distance and angle of both CS and MVA plane and (b) the mitral annulus geometry.

**Methods:**

A total of 215 patients with MSCT and CS to MVA topography evaluation were enrolled in this retrospective study.

**Results:**

This patient cohort included 145 patients without FMR (67.4%, FMR ≤ 1+) and 70 patients (32.6%) with clinically relevant FMR (FMR ≥ 2+). Distance and angulation of CS to MVA planes were highly variable. In all groups, no significant correlation was documented between the distance or angle of CS to MVA planes and left ventricular ejection fraction, left ventricular end-diastolic diameter, or left atrial volume. A significant increase in total CS length could be found in patients with FMR ≥ 2+ compared to the FMR ≤ 1+ group. MVA diameter, area, and perimeter were significantly increased in FMR ≥ 2+ compared to FMR ≤ 1+. In the FMR ≥ 2+ cohort 61% showed a distance of CS to MVA plane <7.8 mm and 58% revealed an angle of CS to MVA plane <14.2°.

**Conclusion:**

Distance and angulation of CS to MVA topography using an MSCT approach are similar between patients with or without FMR, while CS length, MVA area, MVA perimeter, anterior-posterior diameter, and intercommissural diameter are significantly increased in all FMR subgroups. However, ~60% of FMR ≥ 2+ patients showed favorable CS to MVA topography for indirect mitral annuloplasty.

## Introduction

Patient selection plays a crucial role in the success of transcatheter mitral valve interventions since various transcatheter systems are currently available. Indirect annuloplasty using percutaneous mitral valve repair *via* the coronary sinus (CS) is one option to treat functional mitral valve regurgitation (FMR). The Carillon Mitral Contour System (Cardiac Dimensions, Kirkland, WA, USA) showed a reduction of echocardiographic FMR parameters and improved heart failure symptoms in multicenter trials (1–4). Recently, we showed that CS to mitral valve annulus (MVA) topography might predict potential responders to this percutaneous transcatheter intervention (5, 6). This underlines the importance of Multislice CT (MSCT) in patient selection for transcatheter mitral valve interventions. Besides indirect annuloplasty, the transcatheter mitral valve system (MVRx, San Mateo, CA, USA) is effective in decreasing FMR using a CS approach (7). However, little is known about the differences of CS to MVA topography and MVA geometry in patients without FMR in comparison to the various FMR classes (FMR 2+, FMR 3+, and FMR 4+). One might speculate, that there is a change of anatomy and topography with increasing severity of FMR due to more severe left atrial and left ventricular dilation or more advanced reduction in left ventricular ejection fraction. In the absence of FMR, the course of the CS and its topographic relation to the MVA has shown to be highly variable in anatomic studies (8–10). In patients with mitral regurgitation (MR), MSCT revealed an increased CS length and greater distances between the CS and the MVA in comparison to healthy controls (11). However, linear distances from the edge of the MVA to the center of the CS lumen were measured in these studies. This did not reflect the complex anatomical architecture of both structures. We thought to evaluate the anatomical relationship of the CS and the MVA in the absence or presence of FMR using MSCT software, which calculates CS- and MVA-planes to determine distance and angulation of both planes from 3-dimensional reconstructions. We further aimed to determine the percentage of patients with favorable CS to MVA topography for successful indirect mitral valve annuloplasty using the Carillon device in a large cohort of patients with FMR.

Moderate to severe FMR is known to be associated with increased MVA dimensions in MSCT analysis (12). However, no data for FMR subgroups are available yet. Therefore, besides the topographical relationship of CS and MVA in patients with or without FMR, we further aimed to investigate the MVA anatomy using intercommissural (IC) diameter, anterior-posterior (AP) diameter, perimeter, and mitral valve area in patients with and without FMR.

## Materials and Methods

### Patient Cohort

The study was performed according to good clinical practice and in compliance with the Helsinki declaration. An individual written consent was obtained by every patient. Since we performed a retrospective analysis of our patient database, no ethical approval is required due to local regulations. The study comprises 215 patients who underwent MSCT. This retrospective analysis of our hospital database included 145 patients without relevant FMR (FMR ≤ 1+) and 70 patients with clinically relevant FMR ≥ 2+ [VC > 3 mm, regurgitant volume > 30 ml, effective regurgitant orifice area (EROA) > 0.2 cm^2^; FMR ≥ 2+ group]. The FMR ≥ 2+ group contains 33 patients with FMR2+, 24 patients with FMR3+ [VC > 5 mm, regurgitant volume > 45 ml, EROA > 0.3 cm^2^; FMR3+ group], and 13 patients with FMR4+ [VC > 7 mm, regurgitant volume > 60 ml, EROA > 0.4 cm^2^; FMR4+ group].

### MSCT

All patients underwent MSCT for evaluation of coronary artery disease. For MSCT, a 256-slice Brilliance iCT scanner (Philips Healthcare, Amsterdam, Netherlands) was used in accordance with the Society of Cardiovascular CT (SCCT) guidelines (13). A tube current between 200 and 360 mAs at 120 kV, adjusting primarily the mAs based on body habitus. CT scans were performed ECG-gated when applicable in Step and Shoot technique depending on the patient's heart rate and body mass index (BMI). Collimation of CTA was 256 ^*^ 0.6 mm and rotation time 0.27 s. Prior to the scanning protocol, sublingual nitroglycerine spray (800 mcg) was administered to all patients and metoprolol only if necessary (50–150 mg orally 1 h before CT-scan or 5–25 mg intravenously during the scan), aiming for a heart rate <65 beats/min. The scan was triggered using an automatic bolus tracking technique, with a region of interest placed in the descending thoracic aorta and a threshold of 150 Hounsfield units (HU). Prospective ECG-gating was used at 75% of the R-R interval. Nevertheless, persistently elevated heart rates required a retrospective helical protocol in some patients. Contrast agent (Imeron 350, Bracco, Milano, Italy) was administered with a volume of 100 ml (5.7 ml·s-1), followed immediately by a 50 ml saline chaser. Data were reconstructed at 75% of the R-R interval, with a slice thickness of 0.5 mm and a reconstruction interval of 0.3 mm.

**Figure 1:**
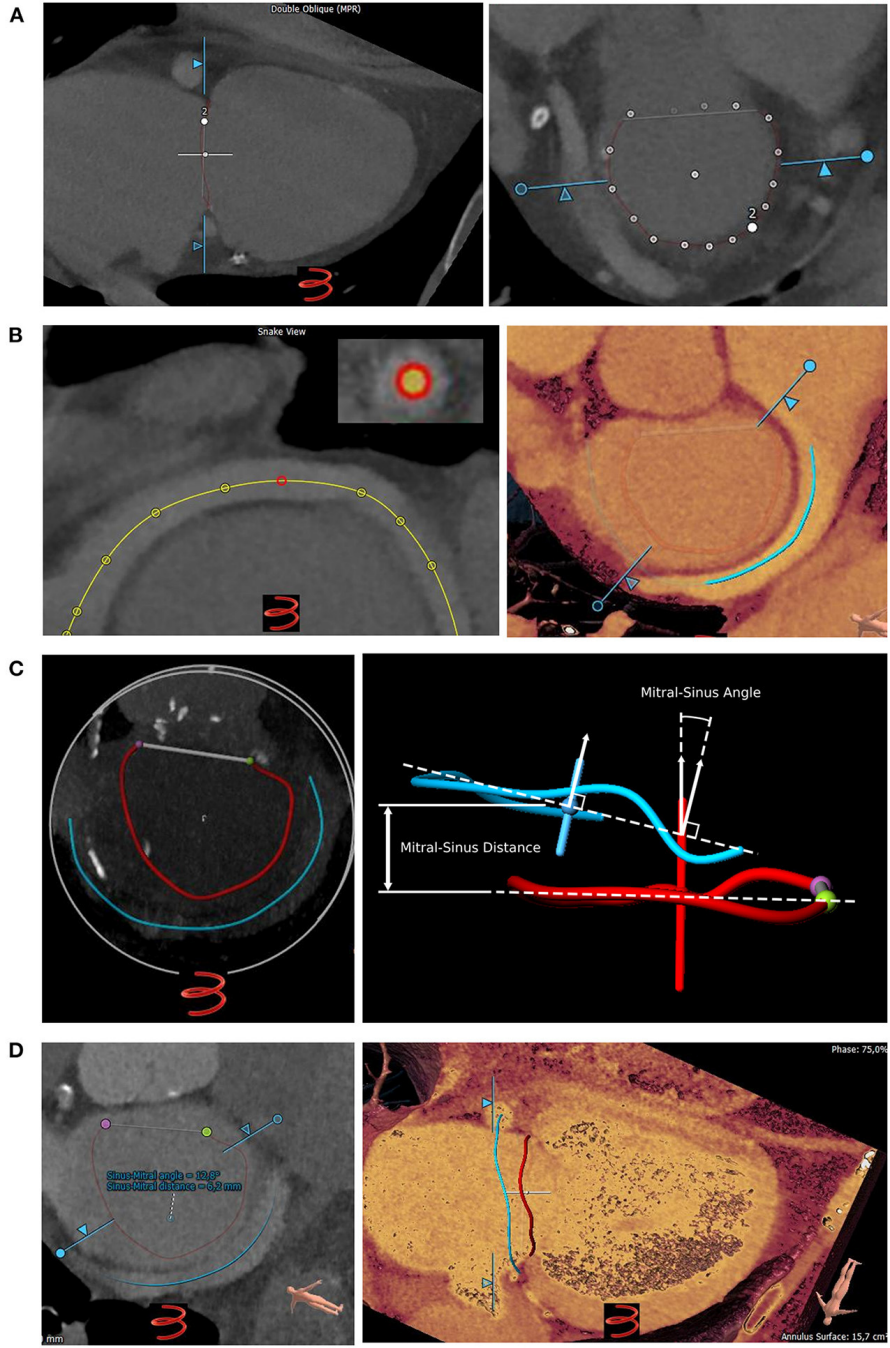
Assessment of coronary sinus to mitral valve annulus topography using a Multislice CT (MSCT) analysis prototype software. **(A)** Left: Semiautomatic tracking of the mitral valve annulus (MVA). Right: White dots indicate the tracked MVA. **(B)** Left: Tracking the coronary sinus (CS; yellow line). Right: MSCT showing the CS (blue line) entering the right atrium. Red line: MVA. **(C)** Left: 3D-model of MVA (red line, D-shape) and CS (blue line) showing their anatomical relation. Right: Centroid (red dot for MVA and blue dot for CS) and axis (red line for MVA and blue line for CS) of the MVA and CS. The normal vector is visualized by the horizontal dotted line. The sinus-mitral angle is located between the axes/normal vectors of the MVA and the CS. **(D)** Left: After MVA and CS reconstruction the prototype software calculates both distance and angulation of CS to MVA planes automatically. Right: Three-dimensional visualization allows evaluation of CS to MVA topography.

The 3mensio Structural Heart (prototype version 10.1) software (Pie Medical Imaging, Maastricht, Netherlands) was used to analyze the MVA using multiplanar reconstructions and volume rendering techniques, as previously described (5, 6). Omission of the anterior peak of the mitral valve (D-shape) was used for MVA analysis. Then, the CS was manually tracked and was visualized in 3D. Distance and angle of the MVA and CS plane were automatically calculated (5, 6). Using eigenvalue decomposition, the normal vector was obtained for the mitral valve and coronary sinus axis. This vector indicated the position and orientation of both planes (CS and MVA). After projecting the centroid of the CS onto the MVA axis, the point on the mitral axis closest to the coronary sinus centroid was used to calculate the mitral-sinus distance. Figure 1 shows the algorithm of the MSCT-analysis in a stepwise approach.

Mitral valve anatomy was assessed by cubic-spline-interpolation of 16 seeding points, which were manually set along with the insertion of the posterior mitral valve leaflet and along the anterior peak, as previously described (12). The lateral and medial fibrous trigones were manually tracked and their distances calculated (TT distance). MVA area and perimeter were calculated by projection onto the least-squares plane fitted to the 3D MVA contour (12). The total annular perimeter was calculated by adding the TT distance to the perimeter. The anterior-posterior (AP) distance was defined as the projected distance from the TT line to the posterior peak and the intercommissural (IC) distance as the diameter perpendicular to the AP distance and parallel to the TT distance transecting the centroid of the MVA (12). MVA dimensions were indexed to BSA to adjust differences in body size. Body surface area (BSA) was calculated from the Mosteller formula (BSA = $\sqrt{\left(weight\;x\;height\right)/3600}$).

### Echocardiography

Transthoracic echocardiography studies were obtained using a Philips iE 33 echocardiography system (Philips, Amsterdam, Netherlands). Vena contracta, proximal isovelocity surface area (PISA), EROA, and regurgitant volume for quantitative mitral valve assessment were recorded according to current recommendations (14). The severity of MR was graded according to a previously reported classification (15). Left ventricular ejection fraction (LVEF) was determined using the Simpsons method in 4- and 2-chamber view.

**Figure 2:**
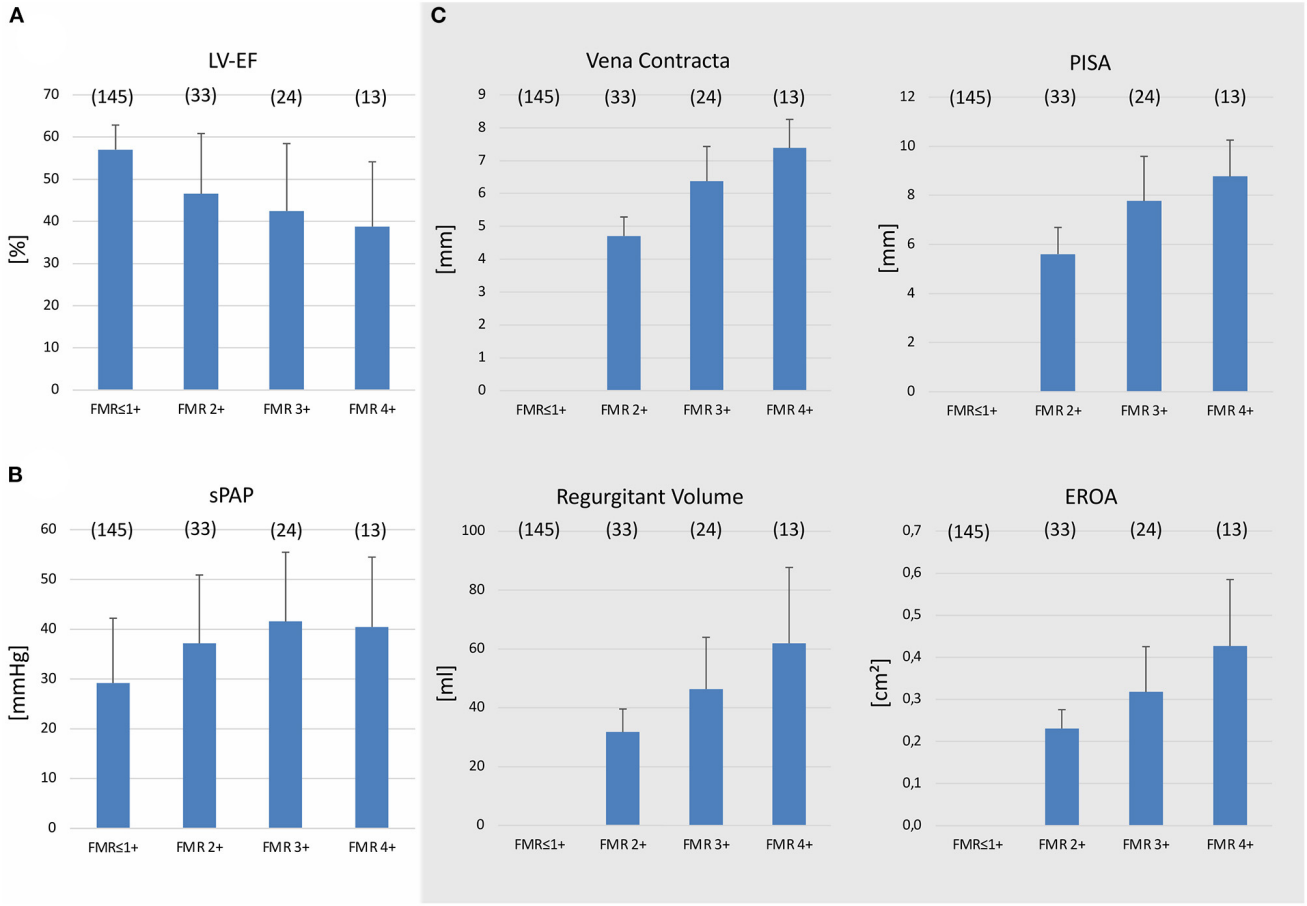
Echocardiographic evaluation of patients with and without functional mitral valve regurgitation. Left ventricular ejection fraction (LVEF) **(A)** and systolic pulmonary artery pressure (sPAP) **(B)** in patients with functional mitral regurgitation (FMR) ≥ 2+ or FMR ≤ 1+. Patients with FMR ≥ 2+ were separated according to a standard classification (FMR 2+, 3+, and 4+). **(C)** Quantitative echocardiographic assessment including measurement of vena contracta, proximal isovelocity surface area (PISA), effective regurgitant orifice area (EROA), and regurgitant volume in patients with FMR ≥ 2+. Mean ± *SD*.

Patients in the FMR ≤ 1+ group showed normal LVEF and systolic pulmonary artery pressure (sPAP) compared to the FMR ≥ 2+ group (Figures 2A,B and Table 1). The FMR subgroups (FMR 2+, FMR3+ and FMR4+) revealed a decreasing LVEF and increasing sPAP with more advanced FMR (Figures 2A,B). Quantitative echocardiographic MR assessment was performed only in the FMR ≥ 2+ group. Patients in the FMR ≥ 2+ group were divided into FMR classes according to these parameters. Vena contracta, PISA, EROA, and regurgitant volume were increasing according to the FMR class (Figure 2C).

**Table 1:** Patients characteristics.

	**FMR≤1+**		**FMR≥2+**		***p*-value**
	***n***	**% or Mean ± SEM**	***n***	**% or Mean ± SEM**	
Age	145	67.6 ± 1.1	70	75.3 ± 0.9	<0.001
Male	67	46.2	36	51.4	0.47
**Patients' history**
Arterial hypertension	112	77.2	56	80.0	0.65
Hyperlipidemia	75	51.7	36	51.4	0.97
Diabetes mellitus	22	15.2	14	20.0	0.37
Coronary artery disease	61	42.1	38	54.3	0.09
Previous heart surgery	11	7.9	20	28.6	<0.001
Ischemic cardiomyopathy	0	0.0	24	34.3	<0.001
Non-ischemic cardiomyopathy	0	0.0	46	65.7	<0.001
Atrial fibrillation	45	31.0	42	60.0	<0.001
Tricuspid regurgitation	5	3.4	27	38.6	<0.001
**Transthoracic echocardiography**
LVEF (%)	145	57.0 ± 0.5	70	44.4 ± 1.8	<0.001
LVEDD (mm)	145	44.1 ± 0.6	70	52.4 ± 1.1	<0.001
sPAP (mmHg)	124	25.6 ± 1.0	68	39.9 ± 1.6	<0.001
LA volume (ml/m^2^)	135	25.2 ± 0.9	49	48.1 ± 2.6	<0.001

*LVEF, left ventricular ejection fraction; LVEDD, left ventricular end diastolic diameter; sPAP, systolic pulmonary artery pressure; LA, left atrium; SEM, standard error of the mean*.

### Statistical Analysis

Statistical analysis was performed using PASW statistics 18 software (SPSS, Chicago, USA). All variables were tested for normal distribution with the Kolmogorov-Smirnov test. In the case of normal distribution, the results are given as SEM if not otherwise indicated, or as median and 95% CI. Differences between groups and subgroups were evaluated by chi-square-test for discrete variables and one-way ANOVA with Scheffe *post-hoc* testing for continuous variables. Pearson correlation coefficient was used for correlations between two variables. For ordinal data, Kruskal-Wallis-Test was used. A *p*-value < 0.05 was considered statistically significant.

## Results

All demographic variables are shown in Table 1. In our patient cohort approximately half of the patients were male (FMR ≤ 1+: 45%, FMR ≥ 2+: 51%) and the mean age accounts for 67.6 ± 1.1 years in patients with FMR ≤ 1+ and 75.3 ± 0.9 years in patients with FMR ≥ 2+. The origin of FMR was non-ischemic (65.7%) or ischemic cardiomyopathy (34.3%).

### Coronary Sinus to Mitral Valve Topography in FMR

**Figure 3:**
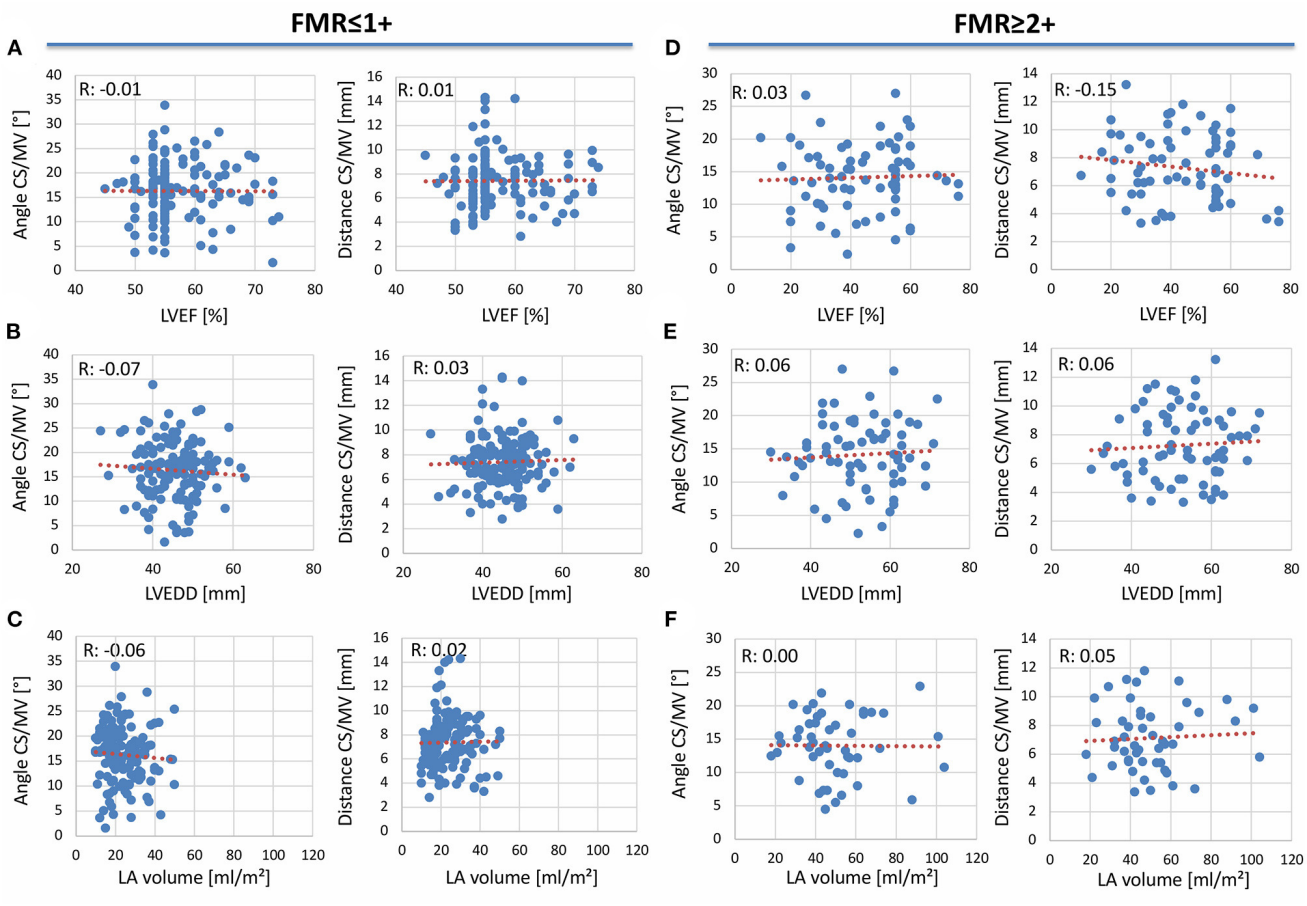
Correlation of angulation and distance of CS to MVA planes with left ventricular ejection fraction left ventricular end-diastolic diameter and left atrial volume in patients with and without FMR. Correlation of angle (left) and distance (right) of the coronary sinus (CS) to mitral valve annulus (MVA) with **(A)** left ventricular ejection fraction (LVEF), **(B)** left ventricular end-diastolic diameter (LVEDD), and **(C)** left atrial (LA) volume in patients with FMR ≤ 1+. Correlation of angulation (left) and distance (right) of CS to MVA with **(D)** LVEF, **(E)** LVEDD, and **(F)** LA volume in patients with FMR ≥ 2+. *R*-values as indicated.

**Figure 4:**
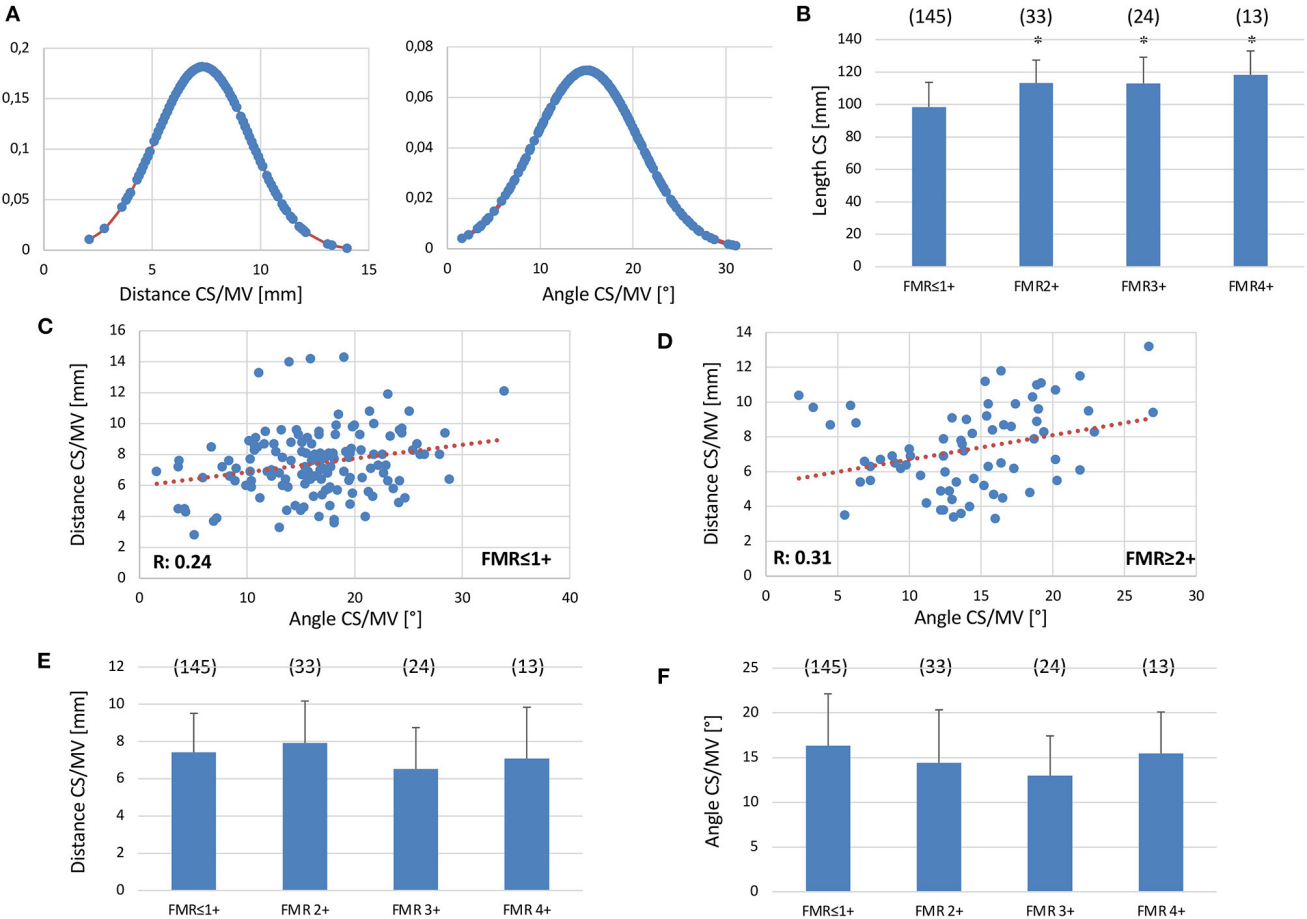
Coronary Sinus to Mitral Valve anatomy and topography in the presence and absence of FMR. **(A)** Gaussian function showing normal distribution of distance (left) and angulation (right) of CS to MVA planes (*n* = 215). **(B)** Total CS length in patients with FMR ≥ 2+ and FMR ≤ 1+. Correlation of distance and angulation of CS to MVA planes of patients with FMR ≤ 1+ **(C)** or FMR ≥ 2+ **(D)**. **(E)** No difference between the distance of CS to MVA in patients with FMR ≤ 1+ or with FMR2+, FMR3+ or FMR4+. **(F)** No difference between angulation of CS to MVA in patients with FMR ≤ 1+ or with FMR2+, FMR3+ or FMR4+. Mean ± SD. *p < 0.05 vs. noFMR.

We used a prototype software for CS reconstruction in MSCT to determine the distance and angle of the MVA and the CS planes in FMR ≤ 1+ and FMR ≥ 2+. To investigate the effect of reduced LV-function with dilation of the left ventricle (LV) and atrium (LA) on these parameters, a correlation of LVEF, left ventricular end-diastolic diameter (LVEDD), and LA-volume with either distance or angle of CS to MVA planes was performed in FMR ≤ 1+ and FMR ≥ 2+. No significant difference could be obtained in any of these groups indicating no relationship between these parameters and the distance or angle of CS to MVA planes (Figure 3). However, both the distance and angle of CS to MVA planes were normally distributed (Figure 4A). A significant increase in total CS length was found in patients with FMR ≥ 2+ in comparison to FMR ≤ 1+ or FMR subgroups (Figure 4B). No direct correlation between distance and angulation of CS to MVA planes could be obtained in FMR ≤ 1+ or FMR ≥ 2+ patients (Figures 4C,D) excluding a direct interaction of both parameters. Distance of CS to MVA planes was 7.42 ± 0.17 mm in FMR ≤ 1+ and 7.28 ± 0.29 mm in FMR ≥ 2+ without a statistically significant difference. Subgroups of FMR showed comparable results (Figure 4E). Angulation of CS to MVA planes showed also no statistically significant difference between the groups and FMR subgroups (Figure 4F).

### Mitral Valve Geometry in FMR

**Figure 5:**
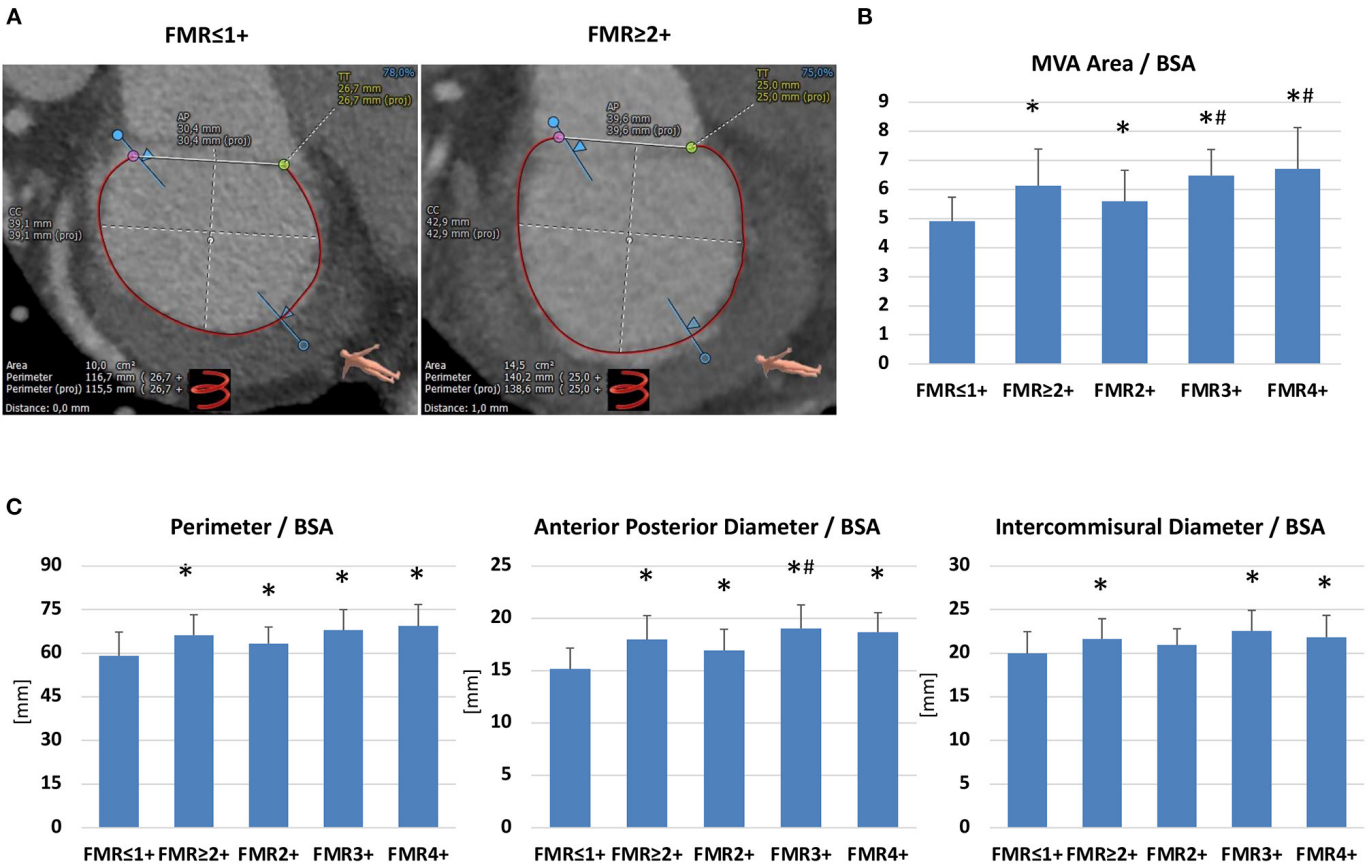
Dimensions of mitral valve annulus dimensions in patients with or without functional mitral regurgitation. **(A)** MSCT-derived parameters of mitral valve annulus dimensions (CC, intercommisural diameter; AP, anterior-posterior diameter; TT, trigone-to-trigone diameter) in a patient with FMR ≤ 1+ and with FMR ≥ 2+. **(B,C)** Mitral Valve Annulus (MVA) area, perimeter, anterior-posterior diameter and intercommisural diameter indexed to the Body Surface Area (BSA) in patients with FMR ≤ 1+ (*n* = 145) compared to FMR ≥ 2+ (*n* = 70) and the FMR subgroups (FMR2 +: *n* = 33, FMR3+: *n* = 24, FMR 4+: *n* = 13). *p <0.05 compared to FMR ≤ 1+; ^#^ *p* <0.05 compared to FMR2+.

All parameters of MVA geometry were markedly increased in patients with FMR ≥ 2+ compared to the FMR ≤ 1+ group (Figure 5A). MVA dimensions were indexed to BSA to adjust differences in body size. However, indexing MVA geometry parameters to BSA showed comparable results for FMR ≥ 2+ and FMR ≤ 1+ (Figures 5B,C). Furthermore, all FMR subgroups showed significantly increased MVA dimension parameters compared to FMR ≤ 1+ (Figures 5B,C). Of note, for MVA area/BSA and perimeter/BSA an increase in these parameters over the subgroups could be obtained, indicating the marked dilated MVA in higher FMR classes (Figures 5B,C).

### Indirect Mitral Valve Annuloplasty

**Figure 6:**
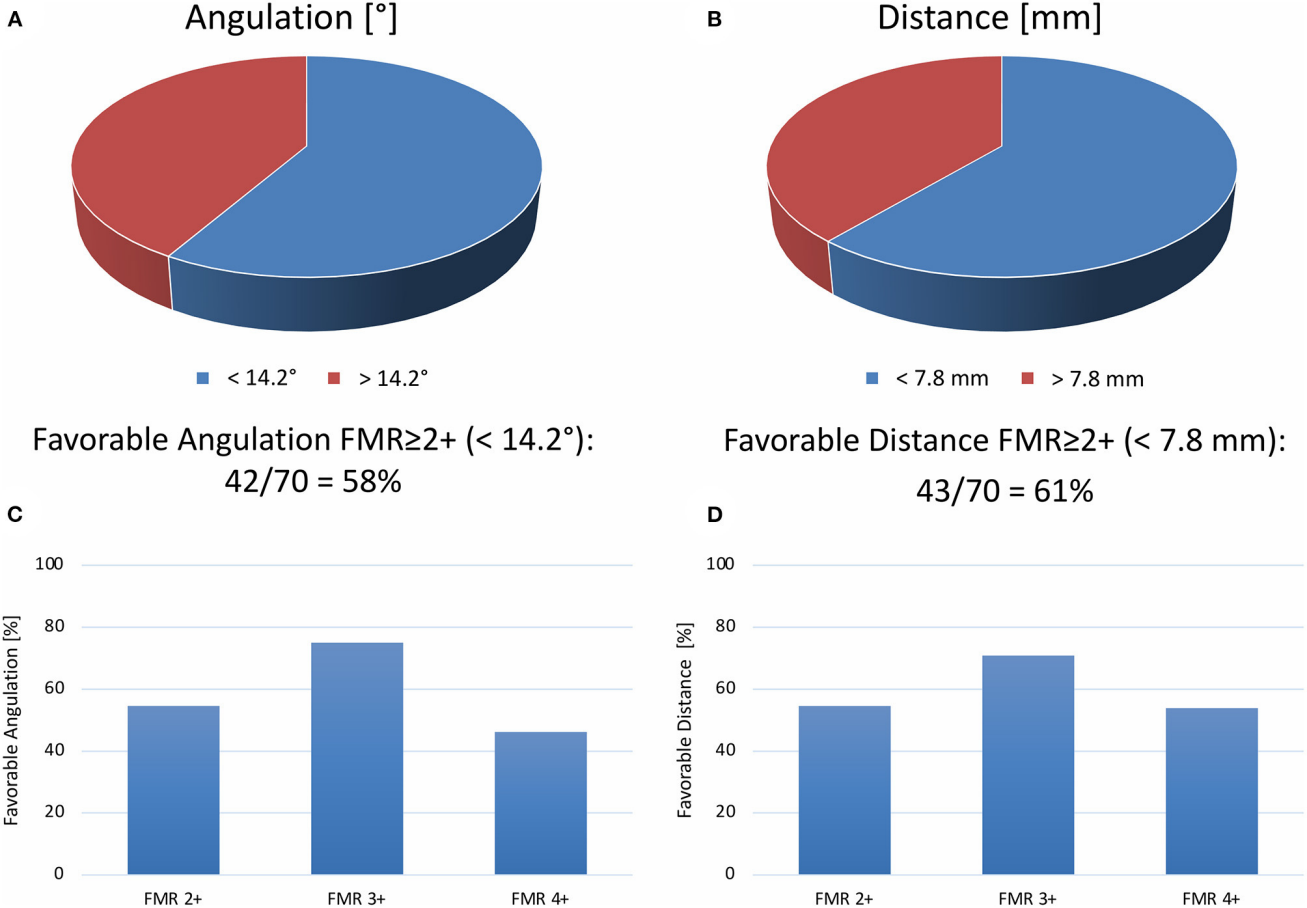
Topography of coronary sinus to mitral valve annulus for percutaneous sinus-based mitral valve annuloplasty. Percentage of patients with favorable angle [ <14.2°, **(A)**] or distance [ <7.8 mm, **(B)**] of the coronary sinus to the mitral valve annulus. Percentage of favorable angle **(C)** or distance **(D)** according to FMR ≥ 2+ classes (FMR2+, FMR3+, or FMR4+).

A CS to MVA topography with a distance of <7.8 mm and an angulation of <14.2° predicts a reduction of FMR after indirect mitral annuloplasty with the Carillon device (5, 6). In the FMR ≥ 2+ cohort, 43 out of 70 patients (61%) showed a distance of CS to MVA plane <7.8 mm, while 42 out of 70 patients (58%) revealed an angle of CS to MVA plane <14.2° (Figures 6A,B). Furthermore, patients with FMR 3+ showed the highest rate of favorable CS to MVA topography and FMR 4+ the lowest rate (Figures 6C,D).

## Discussion

Coronary sinus to MVA topography has been addressed in anatomical studies (8–10). Distances were assessed along the course of the CS and were found to be highly variable. Distances of 5.7 ± 3.3 mm and 9.7 ± 3.2 mm at the level of P2 and P3 segments were reported (10). Furthermore, noninvasive evaluation of CS to MVA anatomy using MSCT has been previously reported in 105 patients (11). The minimal distance was evaluated between CS and MVA and accounted for 5.1 ± 2.9 mm. In patients with more pronounced MR (FMR ≥ 3+), this minimal distance was significantly increased compared to FMR ≤ 2+ (7.3 ± 3.9 mm, *n* = 15 vs. 4.8 ± 2.5 mm, *n* = 90). Also, total CS length was significantly larger in patients with relevant MR (128.6 ± 14.6 mm, *n* = 15 vs. 110.1 ± 16.6 mm, *n* = 90) (11). These MSCT analyses are limited to its minimal distance approach neglecting the fact of highly variable CS distances over the course of the CS. To circumvent this limitation, we worked with MSCT analysis software, which uses the centroid and the normal vector obtained through eigenvalue decomposition of the MVA and CS plane to calculate the distance between the MVA and CS plane.

In contrast to available MSCT data evaluating the CS to MVA relation, we found no significant correlation between neither LV- nor LA-dilation nor reduced LVEF and the distance of CS to MVA planes (11). Furthermore, the angulation of CS to MVA plane did not correlate with LVEDD, LA-volume, or LVEF. CS anatomy and its topography to MVA are not linked to reduced LVEF or cardiac dilation. However, the total CS-length is significantly increased in FMR ≥ 2+ compared to the control group, which is in line with previous results (11).

Indirect mitral annuloplasty *via* the CS using the Carillon Mitral Contour System reduces FMR and improves heart failure symptoms (1–4). The REDUCE-FMR trial is sham-controlled in 135 patients with FMR, comparing CS-based annuloplasty with optimal medical therapy (4). The primary endpoint was met by reducing the regurgitant volume for patients with successful Carillon implantation (4). However, approximately half of the treatment group showed no improvement in FMR after the Carillon device implantation (4). Furthermore, the AMADEUS trial showed no acute improvement on FMR in 26.6% (3). Our previous results showed that shorter distance and lower angulation of the CS to MVA topography influence procedural results (5, 6). A CS plane and MVA plane with a distance of <7.8 mm and an angulation of <14.2° were associated with a reduction of the FMR after Carillon device implantation. Using these cut-off values, 61% of the FMR ≥ 2+ patients in our cohort showed a distance of CS to MVA plane <7.8 mm and 58% revealed an angle of CS to MVA plane <14.2°. This is in accordance with the REDUCE-FMR data, showing half of the patients without improvement of FMR parameters in transthoracic echocardiography. Furthermore, our results indicate that between FMR subgroups or patients without FMR, no significant difference in distance and angulation of CS to MVA plane exists.

Besides indirect mitral annuloplasty, the ARTO device is an emerging technique with promising one-year clinical follow-up (7). This transcatheter technique also uses CS access to reduce FMR. Therefore, one might speculate that distance and angulation of CS to MVA planes might play a role in the degree of FMR reduction following this transcatheter intervention. Further clinical studies are needed to address this question, but our anatomical findings suggest a relevance of these parameters in CS-based FMR procedures.

Besides the CS to MVA topography, we reported D-shaped mitral annulus dimensions for patients with FMR ≤ 1+ or FMR ≥ 2+. As previously reported, we found enlarged MVA dimensions in patients with FMR ≥ 2+. A study on 27 patients with moderate or severe FMR showed MVA area/BSA (FMR ≥ 2+: 6 ± 1.3 vs. FMR ≤ 1+: 4.7 ± 0.6), perimeter / BSA (FMR ≥ 2+: 67 ± 9 vs. FMR ≤ 1+: 59 ± 5), anterior-posterior distance (FMR ≥ 2+: 18.1 ± 3.3 vs. FMR ≤ 1+: 14.8 ± 1.6) and intercommunal distance (FMR ≥ 2+: 21.2 ± 3.1 vs. FMR ≤ 1+: 20.2 ± 1.9) to be markedly increased (12). We confirmed these results in a larger cohort of FMR ≥ 2+ (*n* = 70 patients), which allows us to analyze the different FMR classes separately. All FMR subgroups (FMR 2+, FMR3+, and FMR4+) showed significantly increased MVA dimension parameters compared to FMR ≤ 1+. Notably, for MVA area/BSA and perimeter/BSA an increase in these parameters over the subgroups could be obtained, indicating the marked dilated MVA in higher FMR classes.

In summary, we applied for the first time new MSCT-derived variables (distance and angle of CS to MVA planes) in patients with or without FMR. We provided reference values for CS to MVA distance and CS to MVA angulation. Furthermore, we investigated D-shaped MVA dimensions in subgroups of FMR (FMR2+, FMR3+, and FMR4+) and compared them to controls with FMR ≤ 1+. It is worth noting that we found about 60% of patients with FMR to have favorable CS to MVA topography for mitral valve repair using annuloplasty *via* the CS. Our results show the feasibility of a new non-invasive evaluation of CS to MVA topography and confirmed reference values for D-shaped MVA dimensions in FMR subgroups, which coincide with former studies (11, 12, 16).

## Conclusion

Distance and angulation of CS to MVA topography can be derived from MSCT using a novel approach. This might be helpful for patient selection prior to mitral valve interventions for FMR treatment. We reported 60% of FMR ≥ 2+ patients to have favorable CS to MVA topography for mitral valve repair using indirect annuloplasty *via* the CS. Using the 3D-MSCT derived distance and angle of CS to MVA planes in FMR ≥ 2+ patients can be helpful for device selection prior to mitral valve interventions. This might improve the results of percutaneous mitral valve repair using the Carillon Mitral Contour System.

## Limitations

This is a retrospective, single-center study with consequent statistical limitations. Therefore, the results should be regarded as hypothesis-generating.

## Data Availability Statement

The datasets used and/or analyzed during this study are available from the corresponding author on reasonable request.

## Ethics Statement

Ethical review and approval was not required for the study on human participants in accordance with the local legislation and institutional requirements. The patients/participants provided their written informed consent to participate in this study.

## Author Contributions

DR: conceptualization, data collection, analysis and interpretation, drafting, and final approval. MS: data collection, analysis and interpretation, drafting, and final approval. AÖ: data collection and drafting. HD: conceptualization and critical revision. MH: conceptualization, critical revision, and final approval. All authors contributed to the article and approved the submitted version.

## Conflict of Interest

HD is a consultant for Biotronik and Cardiac Dimensions. MH is a consultant for Biotronik, Orbus Neich, Robocath and Cardiac Dimensions. He received institutional grants and lecture fees from Biotronik, Cardiac Dimensions, Orbus Neich, SMT and Philips. The remaining authors declare that the research was conducted in the absence of any commercial or financial relationships that could be construed as a potential conflict of interest.

## Publisher's Note

All claims expressed in this article are solely those of the authors and do not necessarily represent those of their affiliated organizations, or those of the publisher, the editors and the reviewers. Any product that may be evaluated in this article, or claim that may be made by its manufacturer, is not guaranteed or endorsed by the publisher.
